# Supplementary material for: Trajectories of plasma and CSF MTBR-tau243 and phosphorylated-tau species across the Alzheimer’s disease continuum
Source: Nat Commun. 2026 Apr 9;17:3400. doi: 10.1038/s41467-026-71732-1 (PMC13065743; doi:10.1038/s41467-026-71732-1)
Supplement: Supplementary file 1 — Supplementary Information [file 41467_2026_71732_MOESM1_ESM.pdf]

Supplementary materials

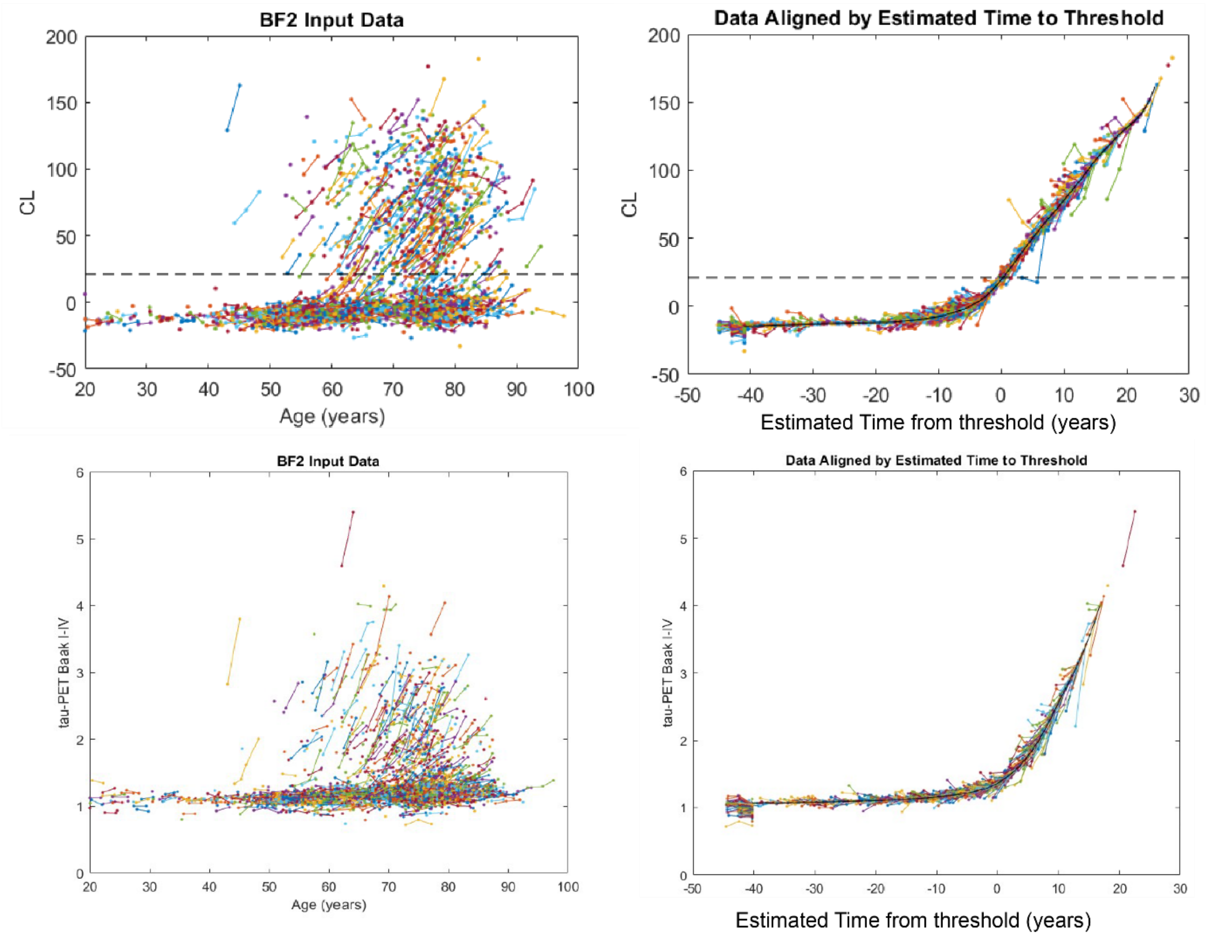

Supplementary Figure-1. SILA model fit amyloid- and tau-PET

Spaghetti plots illustrate the raw Aβ-PET (Centiloid [CL]) and tau-PET data (temporal meta-ROI) in the left panels, and the SILA fitted data arranged by time in the right panels.

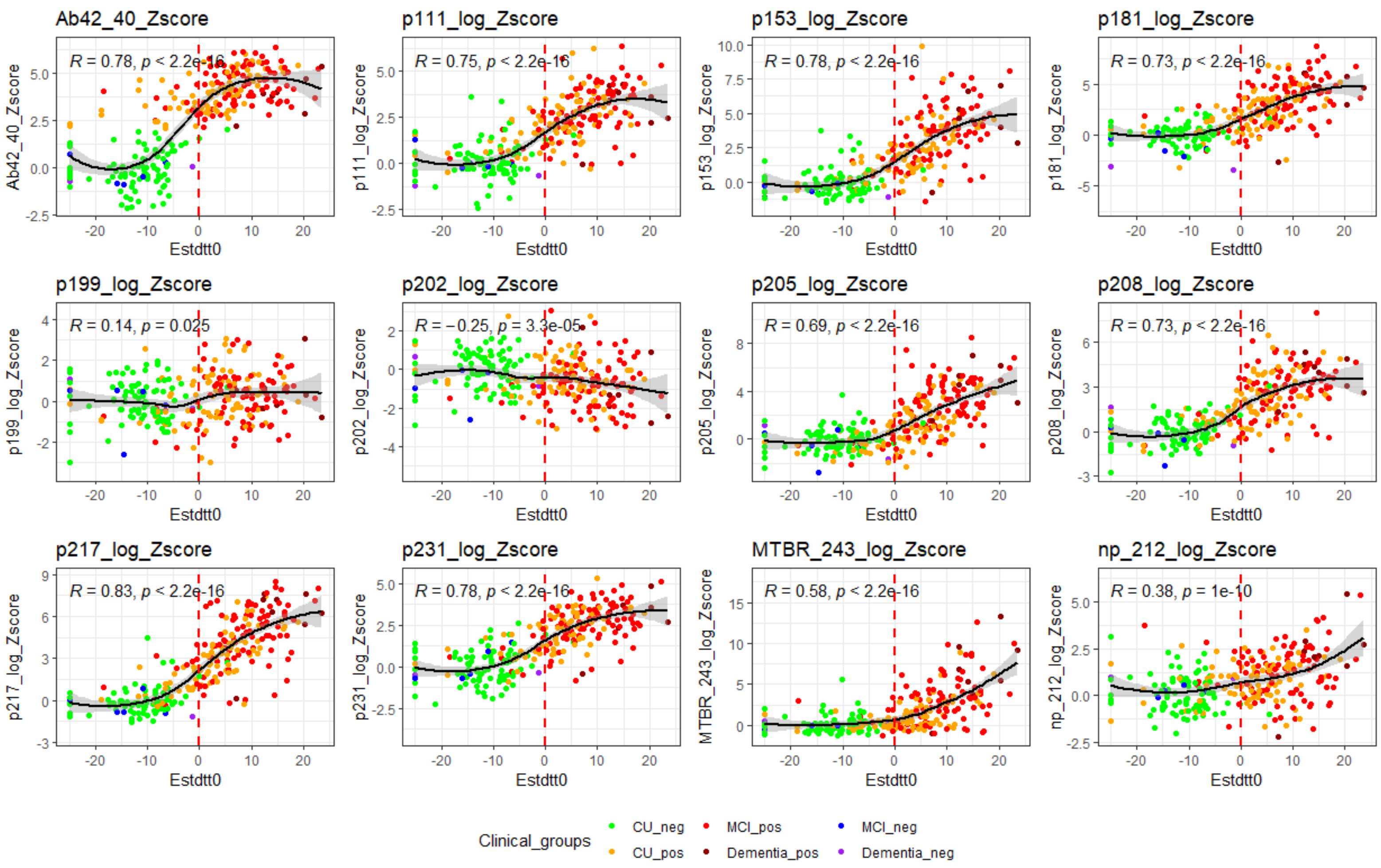

Supplementary Figure-2. Aβ-PET chronicity vs CSF p-tau forms, np-tau mid-region, and MTBR-tau 243

Scatterplots with LOESS curve fits illustrate the behavior of CSF tau biomarkers across amyloid-PET chronicity. Dots are color coded for clinical groups, which is a combination of clinical diagnosis and CSF Aβ42/40 status. Note, that all available cases were included, also MCI and dementia patients who experienced cognitive complaints due to other causes than AD (*i.e.,* MCI_neg and Dementia_neg). Dashed red line is moment of amyloid-PET onset (20 CL). R is the spearman correlation. Gray-shaded areas reflect the 95% confidence interval.

A)

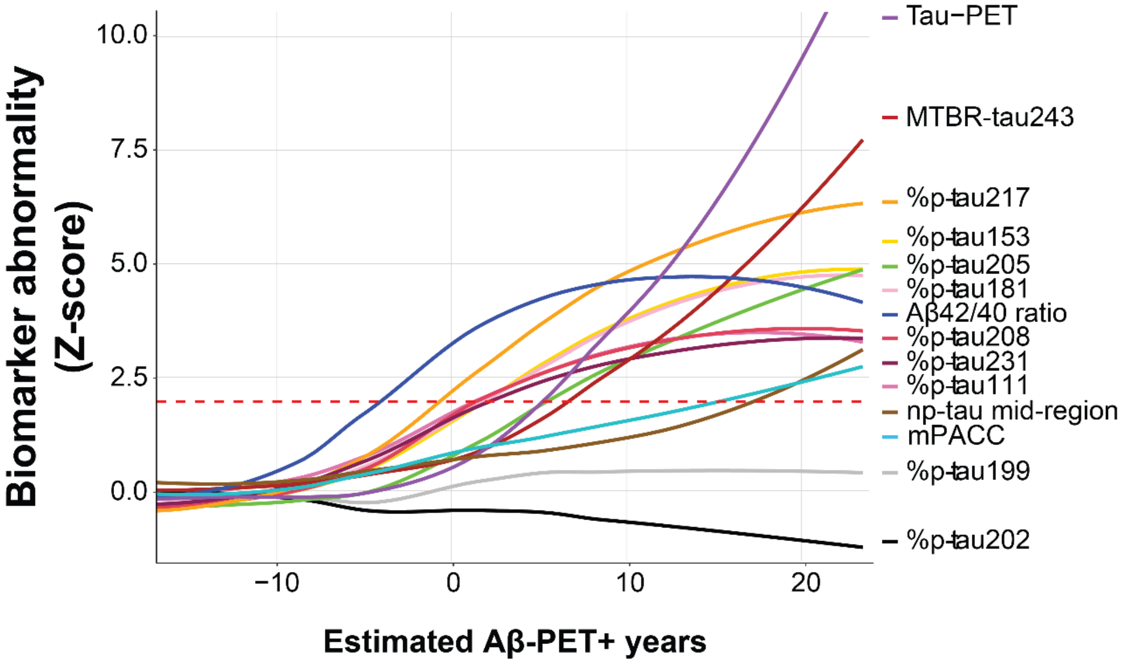

B)

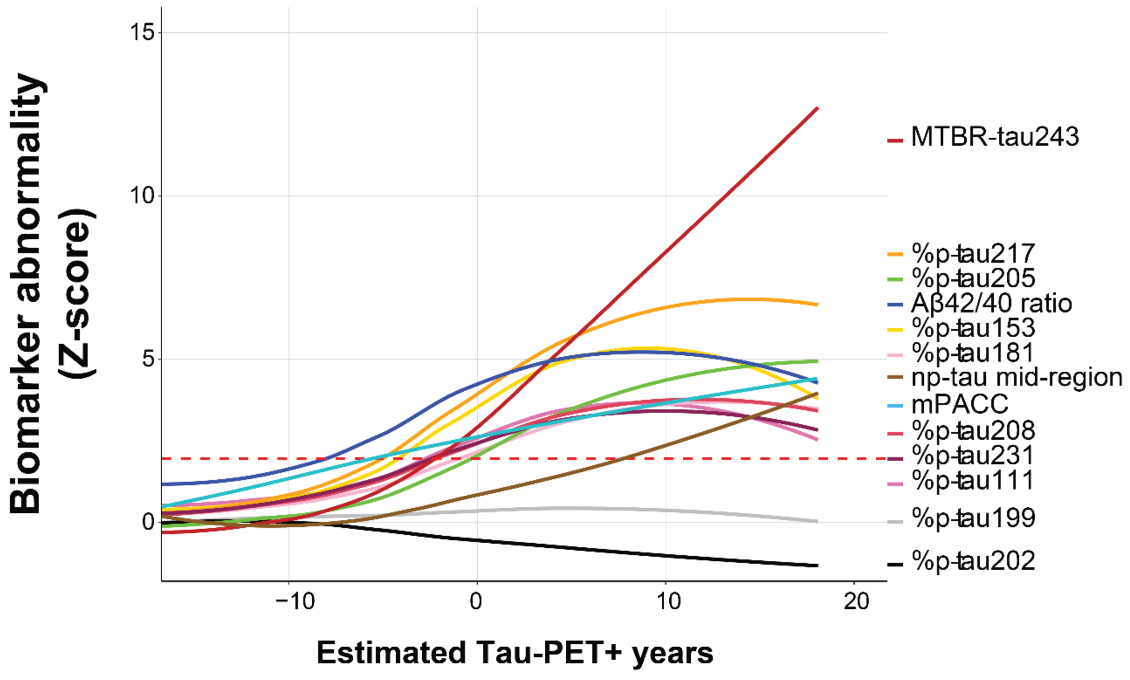

Supplementary Figure-3. CSF biomarker abnormality against AD-pathological PET chronicity
All CSF biomarker abnormality against **A)** Aβ-PET chronicity, with onset (x-axis=0) set at CL=20 and **B)** tau-PET chronicity, with onset (x-axis=0) set at temporal META-ROI = 1.36 SUVR. PET-positivity onset is indicated with the black dotted line. Panels illustrate changes in z-scores, based on the mean and standard deviation of the cognitively unimpaired amyloid negative cases. Shaded area's indicate the 95% confidence interval. Red dotted line for z-score figures illustrates the 2SD (Z-score=1.96) from the reference population.

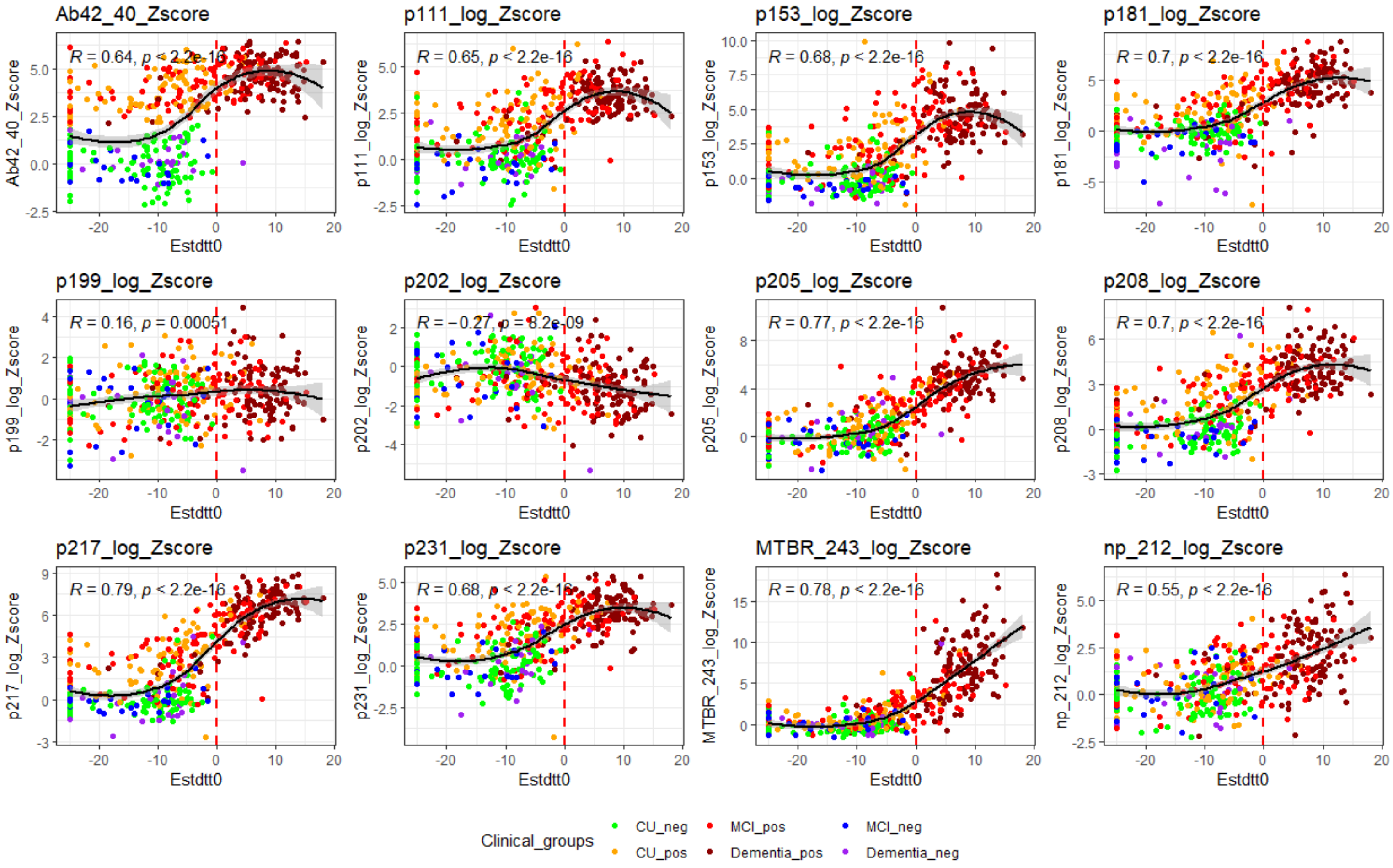

Supplementary Figure-4. Tau-PET chronicity vs CSF p-tau forms, np-tau mid-region, and MTBR-tau 243

Scatterplots with LOESS curve fits illustrate the behavior of CSF tau biomarkers across amyloid-PET chronicity. Dots are color coded for clinical groups, which is a combination of clinical diagnosis and CSF Aβ42/40 status. Note, that all available cases were included, also MCI and dementia patients who experienced cognitive complaints due to other causes than AD (*i.e.,* MCI_neg and Dementia_neg). Dashed red line is moment of amyloid-PET onset (20 CL). R is the spearman correlation. Gray-shaded areas reflect the 95% confidence interval.

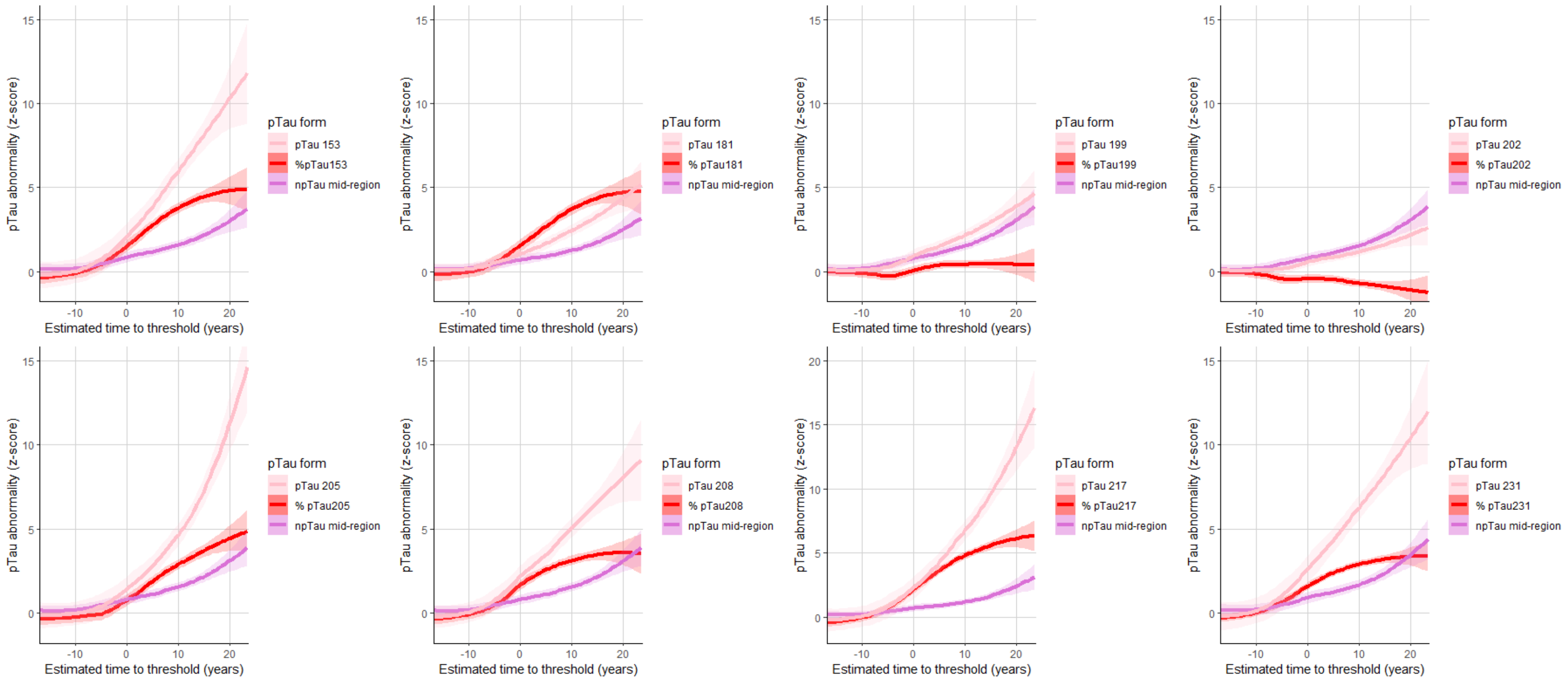

Supplementary Figure-5. CSF p-tau forms across Aβ-PET chronicity

Plots with LOESS fit demonstrate changes in p-tau, %p-tau%, and np-tau mid-regions across tau species. Note, that all np-tau mid-regions reference epitope is specific to each p-tau species. Shaded areas reflect the 95% confidence interval.

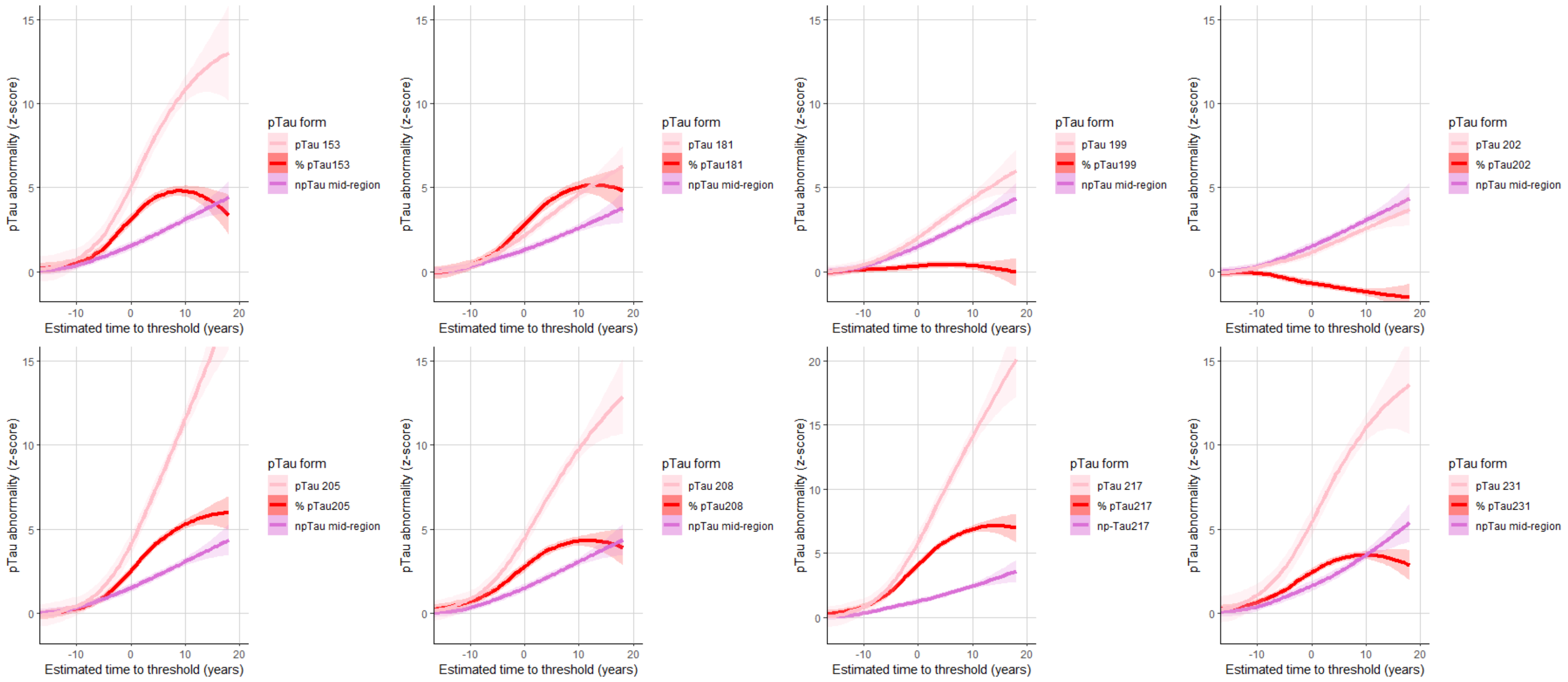

Supplementary Figure-6. CSF p-tau forms across tau-PET chronicity

Plots with LOESS fit demonstrate changes in p-tau, %p-tau%, and np-tau mid-regions across tau species. Note, that all np-tau mid-regions reference epitope is specific to each p-tau species. Shaded areas reflect the 95% confidence interval.

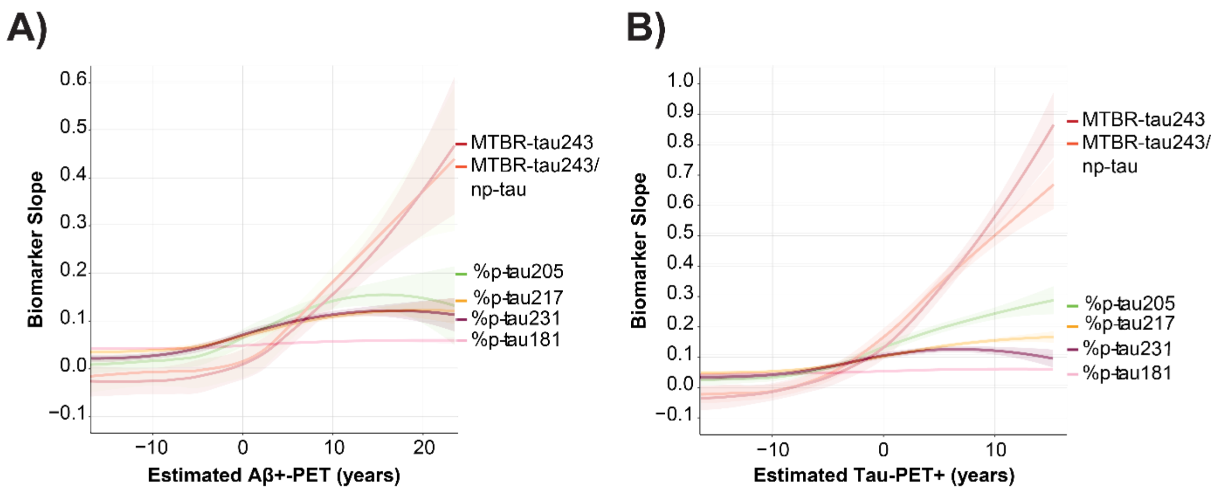

Supplementary Figure-7. Longitudinal CSF p-tau 181 and 231 forms
CSF biomarker abnormality against Aβ-PET chronicity, with onset set at CL=20 and tau-PET chronicity, with onset set at temporal META-ROI = 1.36 SUVR. PET-positivity onset is indicated with the black dotted line. **A/B)** illustrates slope in 181 and 231 %p-tau forms vs amyloid- and tau-PET chronicity, respectively. Trajectories of key markers are shown in the background. Shaded area's indicate the 95% confidence interval. Shaded areas reflect the 95% confidence interval.

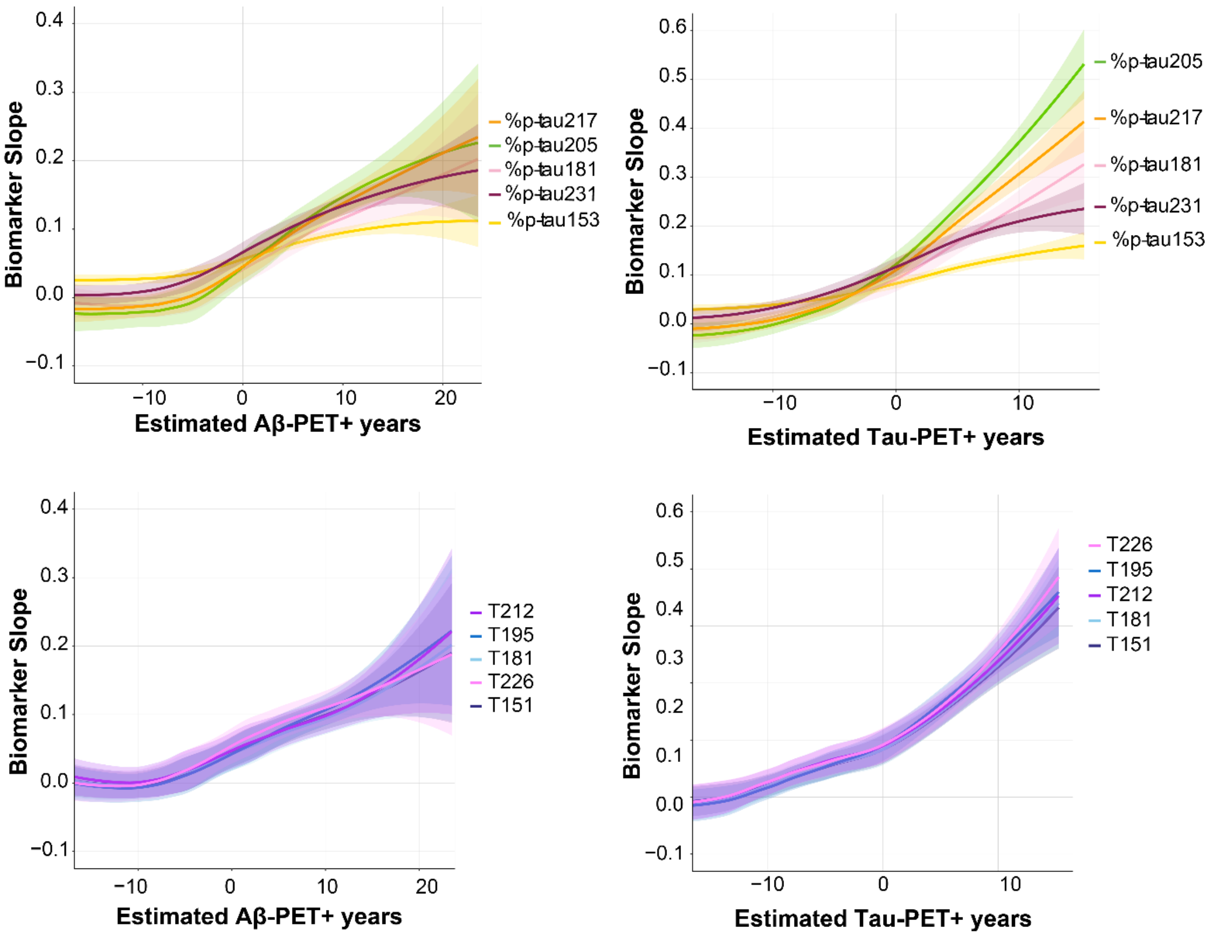

Supplementary Figure-8. Longitudinal Slope CSF p-tau forms across chronicity measures

Plot illustrates LOESS fits of the longitudinal p-tau (top panel) and non-phosphorylated mid-region tau (bottom panel) trajectories. Shaded areas reflect the 95% confidence interval.

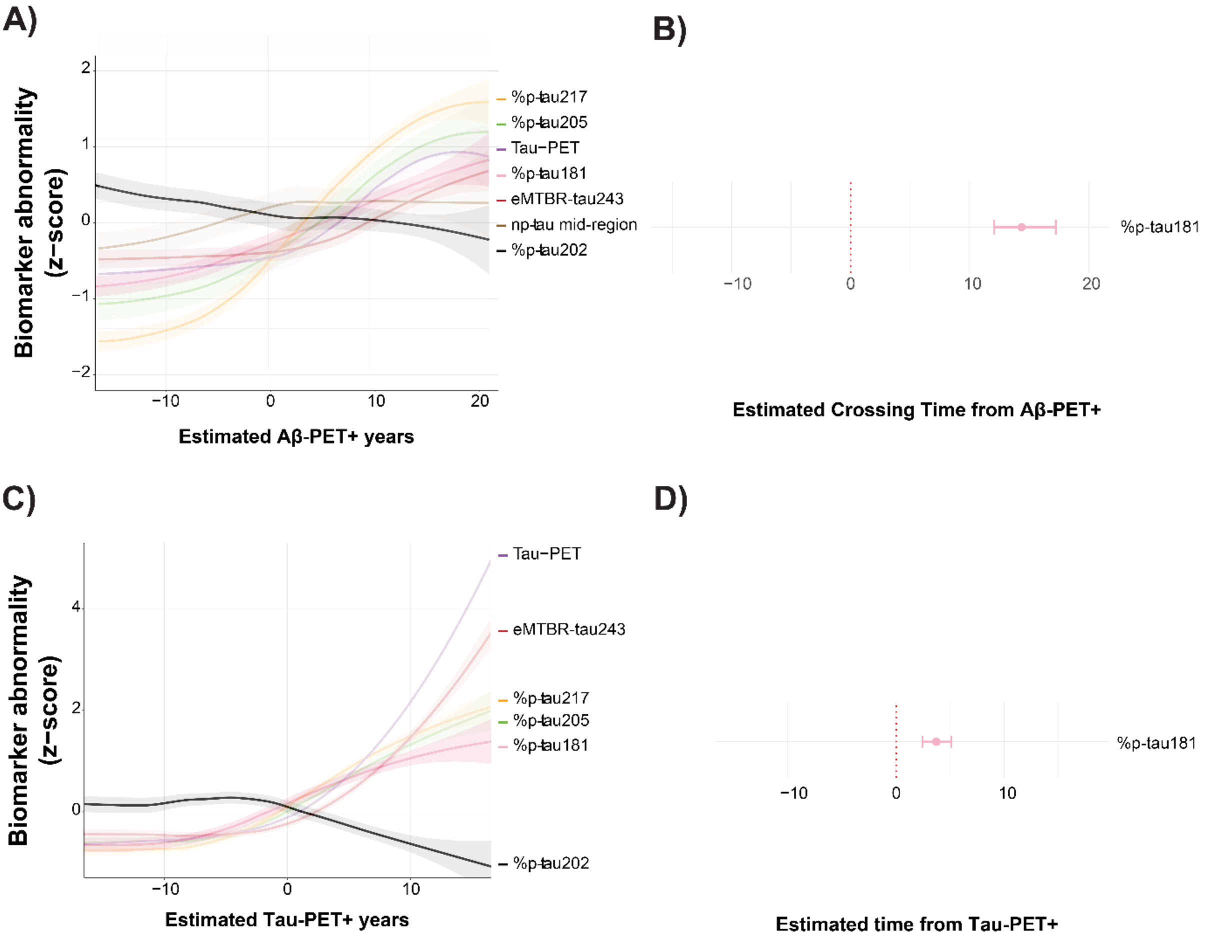

Supplementary Figure-9. Plasma 181 and 202 biomarker abnormality vs AD-pathological PET chronicity

Plasma biomarker abnormality against **A/B)** Aβ-PET chronicity, with onset set at CL=20 and **C/D)** tau-PET chronicity, with onset set at temporal META-ROI = 1.36 SUVR. PET-positivity onset is indicated with the black dotted line. **A/C** illustrate changes in z-scores, based on the mean and standard deviation of the whole population. Shaded area's indicate the 95% confidence interval. **B/D** Forest plots demonstrate the point of p-tau 181 biomarker crossing the 2SD (Z-score=1.96) from the reference population, with 0 (red dotted line) indicated amyloid/tau-PET positivity, respectively. Note, that %p-tau202 is not included in the forest plot, as it did not reach sufficient biomarker abnormality at any point of amyloid-/tau-PET chronicity (*n*=576 and *n*=784 for amyloid and tau, respectively).

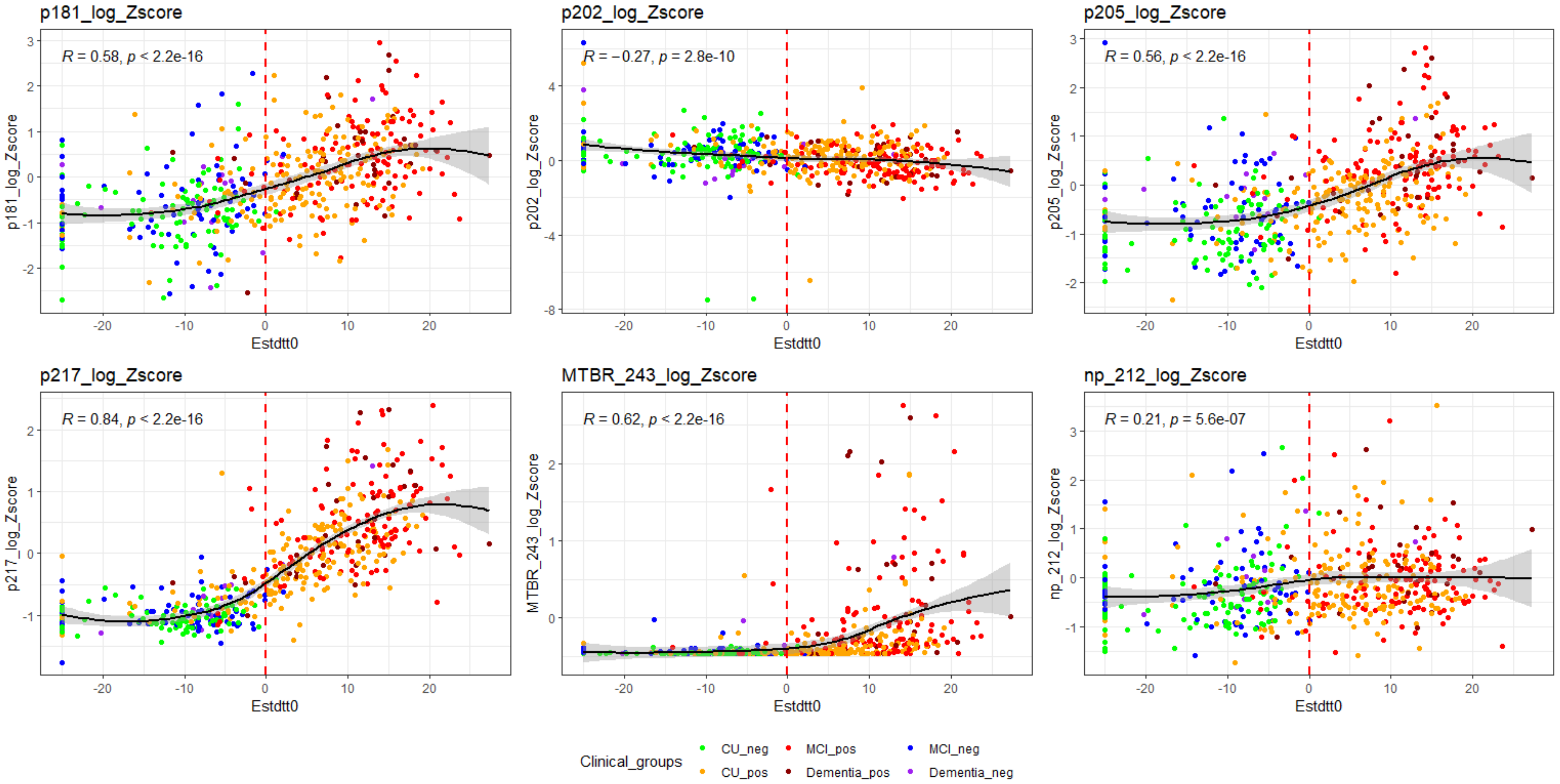

Supplementary Figure-10. Aβ-PET chronicity vs plasma p-tau forms, np-tau mid-region, and MTBR-tau 243

Scatterplots with LOESS curve fits illustrate the behavior of CSF tau biomarkers across amyloid-PET chronicity. Dots are color coded for clinical groups, which is a combination of clinical diagnosis and CSF Aβ42/40 status. Note, that all available cases were included, also MCI and dementia patients who experienced cognitive complaints due to other causes than AD (*i.e.*, MCI_neg and Dementia_neg). Dashed red line is moment of amyloid-PET onset (20 CL). R is the spearman correlation. Gray-shaded areas reflect the 95% confidence interval.

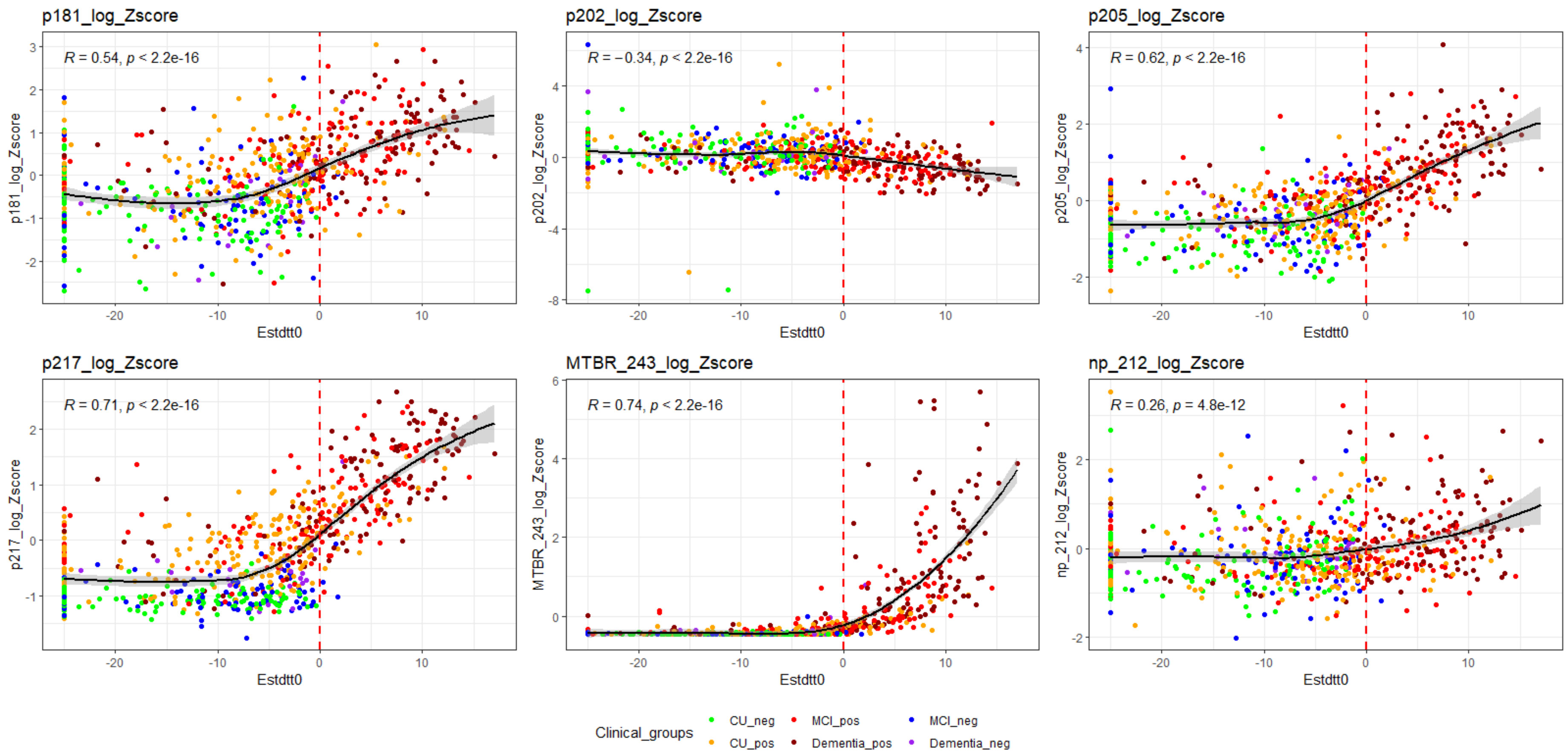

Supplementary Figure-11. Tau-PET chronicity vs plasma p-tau forms, np-tau mid-region, and MTBR-tau 243

Scatterplots with LOESS curve fits illustrate the behavior of CSF tau biomarkers across amyloid-PET chronicity. Dots are color coded for clinical groups, which is a combination of clinical diagnosis and CSF Aβ42/40 status. Note, that all available cases were included, also MCI and dementia patients who experienced cognitive complaints due to other causes than AD (*i.e.,* MCI_neg and Dementia_neg). Dashed red line is moment of amyloid-PET onset (20 CL). R is the spearman correlation. Gray-shaded areas reflect the 95% confidence interval.

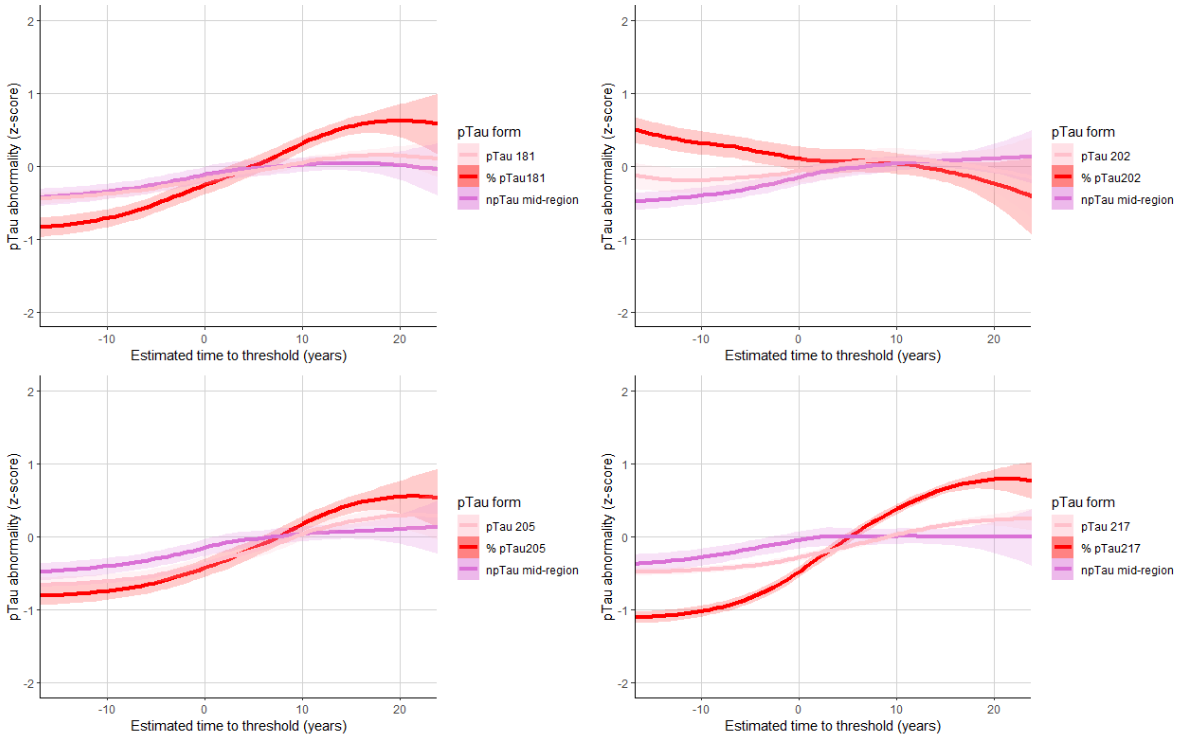

Supplementary Figure-12. Plasma p-tau forms across Aβ-PET chronicity
Plots with LOESS fit demonstrate changes in p-tau, %p-tau%, and np-tau mid-regions across tau species. Note, that all np-tau mid-regions reference epitope is specific to each p-tau species. Shaded areas reflect the 95% confidence interval.

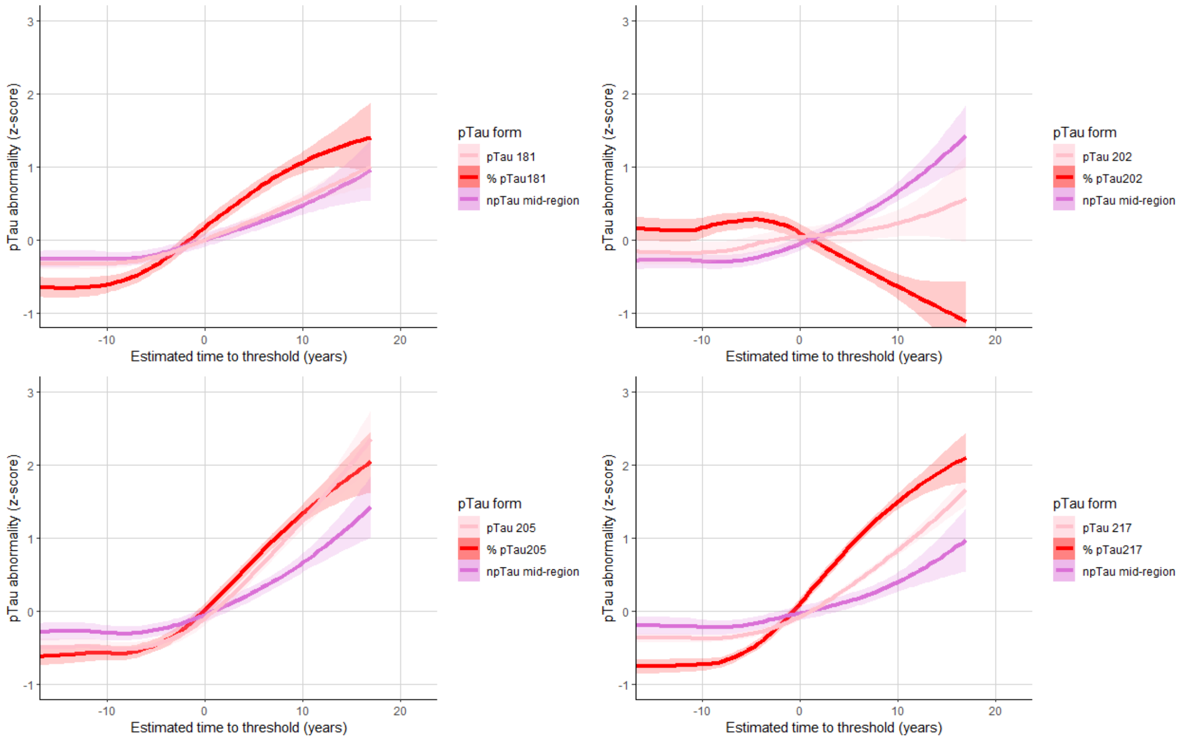

Supplementary Figure-13. CSF p-tau forms across tau-PET chronicity

Plots with LOESS fit demonstrate changes in p-tau, %p-tau%, and np-tau mid-regions across tau species. Note, that all np-tau mid-regions reference epitope is specific to each p-tau species. Shaded areas reflect the 95% confidence interval.

Supplementary Table-1. Stratified analysis for biological sex and *APOE*-Ɛ4 carriership in CSF biomarker trajectories

Biomarker	Aβ-PET+				Tau-PET+			
	Males	***Females***	***APOE-Ɛ4 non-carrier***	***APOE-Ɛ4 carrier***	***Males***	***Females***	***APOE-Ɛ4 non-carrier***	***APOE-Ɛ4 carrier***
**%p-tau217**	-0.51 (-2.06-1.06)	-1.01 (-2.55-0.63)	1.42 (0.06-2.97)	-1.87 (-3.52- -0.36)	-5.32 (-6.63- -3.86)	-5.16 (-6.64- -4.03)	-3.90 (-5.19- -2.63)	-7.65 (-10.84- -5.66)
**%p-tau111**	1.14 (-0.60-2.85)	1.54 (-0.46-4.54)	3.33 (1.67-5.24)	-0.22 (-2.35-1.82)	-2.55 (-4.28- -1.17)	-2.74 (-4.27- -1.21)	-1.47 (-2.91-0.05)	-4.11 (-7.73- -1.86)
**%p-tau208**	1.63 (-0.80-4.06)	1.26 (-0.64-4.69)	4.87 (1.24-8.78)	0.27 (-1.28-2.31)	-2.73 (-4.87- -1.25)	-3.22 (-4.84- -1.77)	-2.00 (-3.65- -0.33)	-4.80 (-7.73- -2.64)
**%p-tau153**	1.24 (-0.60-3.28)	2.81 (0.54-.5.08)	3.15 (1.33-5.73)	0.95 (-0.93.3.58)	-3.33 (-4.89- -1.86)	-3.31 (-4.76- -1.89)	-2.23 (-3.82- -0.78)	-4.62 (-7.13- -2.66)
**%p-tau181**	1.92 (-0.90-3.87)	1.63 (-0.57-4.72)	4.87 (2.76-6.69)	-0.60 (-3.23-1.92)	-2.81 (-4.95- -0.74)	-3.06 (-4.36- -1.69)	-1.09 (-2.76-0.81)	-5.14 (-7.50- -2.81)
**%p-tau231**	1.63 (-0.36-3.77)	2.90 (1.07-4.99)	3.51 (1.54-6.39)	1.24 (-0.99-3.70)	-2.73 (-4.24- -1.23)	-2.50 (-3.87- -1.13)	-1.32 (-2.96-0.35)	-4.02 (-6.45- -2.04)
**%p-tau205**	6.59 (3.28-9.60)	4.90 (2.63-6.81)	8.87 (6.14.11.87)	4.15 (1.82-6.53)	-1.25 (-2.38- -0.22)	-2.33 (-3.55- -1.05)	-0.40 (-1.84-1.30)	-2.21 (-3.42- -1.35)
**MTBR-tau243**	8.43 (5.49-11.83)	5.63 (3.90-7.53)	8.14 (6.33-11.79)	6.20 (-25.00-8.91)	-1.77 (-3.18- -0.04)	-1.77 (-2.79- -0.62)	-0.25 (-1.54-1.34)	-2.73 (-3.92- -1.35)
**mPACC**	15.82 (13.45-20.20)	12.44 (7.90-17.75)	9.23 (5.55-14.96)	16.89 (13.97-21.00)	-0.05 (-2.04-2.28)	1.85 (0.09-3.21)	-0.63 (-1.84-1.30)	1.50 (-0.05- 2.88)
**np-tau mid-region**	23.11 (-25.00-23.40)	14.17 (10.90-17.87)	19.32 (11.12-20.04)	18.25 (-25.00-22.63)	7.63 (4.64-10.85)	4.92 (2.90-7.01)	6.96 (4.68-9.09)	5.82 (3.47-8.06)

Biomarkers are ordered based on abnormality onset of whole CSF biomarker data-set.
Data is presented as bootstrapped onset point and (95% confidence interval)

Supplementary Table-2. Stratified analysis for biological sex and *APOE*-Ɛ4 carriership in Plasma biomarker trajectories

Biomarker	Tau-PET+			
	Males	***Females***	***APOE-Ɛ4 non-carrier***	***APOE-Ɛ4 carrier***
**%p-tau217**	-7.72 (-25.00- -5.63)	-7.05 (-25.00-5.70)	-3.99 (-25.00- -2.87)	-22.70 (-25.00- -10.30)
**MTBR-tau243**	-3.92 (-4.79- -2.57)	-3.51 (-25.00- -1.41)	-3.99 (-5.52- -2.06)	-3.52 (-22.15- -2.49)
**%p-tau205**	1.95 (0.75-3.14)	1.89 (1.06-2.85)	2.69 (1.27-4.06)	1.55 (0.76-2.42)
**mPACC**	4.96 (3.13-6.78)	3.34 (1.97-5.19)	4.46 (1.86-7.04)	3.69 (2.34-5.06)

Biomarkers are ordered based on abnormality onset of whole plasma biomarker data-set.
Note, np-tau mid-region could not be robustly estimated in subpopulation and is therefore excluded from the table
Data is presented as bootstrapped onset point and (95% confidence interval)
