# Supplementary material for: Trajectories of plasma and CSF MTBR-tau243 and phosphorylated-tau species across the Alzheimer’s disease continuum
Source: Nat Commun. 2026 Apr 9;17:3400. doi: 10.1038/s41467-026-71732-1 (PMC13065743; doi:10.1038/s41467-026-71732-1)
Supplement: Supplementary file 2 — Reporting Summary [file 41467_2026_71732_MOESM2_ESM.pdf]

nature portfolio

Corresponding author(s): Lyduine E. Collij

Last updated by author(s): Mar 25, 2026

Reporting Summary

Nature Portfolio wishes to improve the reproducibility of the work that we publish. This form provides structure for consistency and transparency in reporting. For further information on Nature Portfolio policies, see our Editorial Policies and the Editorial Policy Checklist.

Statistics

For all statistical analyses, confirm that the following items are present in the figure legend, table legend, main text, or Methods section.

n/a	Confirmed	
☐	☒	The exact sample size (*n*) for each experimental group/condition, given as a discrete number and unit of measurement
☐	☒	A statement on whether measurements were taken from distinct samples or whether the same sample was measured repeatedly
☐	☒	The statistical test(s) used AND whether they are one- or two-sided<br>*Only common tests should be described solely by name; describe more complex techniques in the Methods section.*
☒	☐	A description of all covariates tested
☐	☒	A description of any assumptions or corrections, such as tests of normality and adjustment for multiple comparisons
☐	☒	A full description of the statistical parameters including central tendency (e.g. means) or other basic estimates (e.g. regression coefficient) AND variation (e.g. standard deviation) or associated estimates of uncertainty (e.g. confidence intervals)
☐	☒	For null hypothesis testing, the test statistic (e.g. *F*, *t*, *r*) with confidence intervals, effect sizes, degrees of freedom and *P* value noted<br>*Give P values as exact values whenever suitable.*
☒	☐	For Bayesian analysis, information on the choice of priors and Markov chain Monte Carlo settings
☒	☐	For hierarchical and complex designs, identification of the appropriate level for tests and full reporting of outcomes
☐	☒	Estimates of effect sizes (e.g. Cohen's *d*, Pearson's *r*), indicating how they were calculated

Our web collection on statistics for biologists contains articles on many of the points above.

Software and code

Policy information about availability of computer code

Data collection	BioFINDER-2 was approved by the Ethical Review Board in Lund, Sweden, which is part of the Swedish Ethical Review Authority (#2016-1053).
Data analysis	The SILA algorithm is freely available at GitHub (https://github.com/Betthauser-Neuro-Lab/SILA-AD-Biomarker).

For manuscripts utilizing custom algorithms or software that are central to the research but not yet described in published literature, software must be made available to editors and reviewers. We strongly encourage code deposition in a community repository (e.g. GitHub). See the Nature Portfolio guidelines for submitting code & software for further information.

Data

Policy information about availability of data

All manuscripts must include a data availability statement. This statement should provide the following information, where applicable:

- Accession codes, unique identifiers, or web links for publicly available datasets
- A description of any restrictions on data availability
- For clinical datasets or third party data, please ensure that the statement adheres to our policy

Provide your data availability statement here.

Research involving human participants, their data, or biological material

Policy information about studies with human participants or human data. See also policy information about sex, gender (identity/presentation), and sexual orientation and race, ethnicity and racism.

Reporting on sex and gender	Included in first paragraph of the results section and stratified analysis for biological sex provided.
Reporting on race, ethnicity, or other socially relevant groupings	n/a
Population characteristics	The CSF cohort (n=446) consisted of 183 cognitively unimpaired participants and 263 cognitively impaired patients (mild cognitive impairment [MCI]: n=124; dementia: n=139), of which 219 (49.1%) were females, 258 (57.8%) were APOE-Ɛ4 carriers, and the mean age at baseline was 71.4 (±8.50) years old. The plasma cohort (n=784) consisted of 331 cognitively normal and 453 impaired patients (MCI n=245; dementia: n=208), of which 398 (50.8%) were females, 477 (60.8%) were APOE-Ɛ4 carriers, with a mean age of 72.2 (±9.27) years. (reported first paragraph of results section)
Recruitment	Participants were recruited at Skåne University Hospital and the Hospital of Ängelholm in Sweden and enrolled between April 2017 and March 2022.
Ethics oversight	This manuscript used data from the BioFINDER-2 cohort. BioFINDER-2 was approved by the Ethical Review Board in Lund, Sweden, which is part of the Swedish Ethical Review Authority (#2016-1053).

Note that full information on the approval of the study protocol must also be provided in the manuscript.

Field-specific reporting

Please select the one below that is the best fit for your research. If you are not sure, read the appropriate sections before making your selection.

[x] Life sciences [ ] Behavioural & social sciences [ ] Ecological, evolutionary & environmental sciences

For a reference copy of the document with all sections, see nature.com/documents/nr-reporting-summary-flat.pdf

Life sciences study design

All studies must disclose on these points even when the disclosure is negative.

Sample size	All individuals with available mass-spec CSF (n=446) and plasma (n=784) biomarkers were included. No participants were excluded.
Data exclusions	Included data within 1 year of primary measurement (CSF or plasma marker); this is stated in the methods section
Replication	Analysis were performed bootstrapped (1000 interations) to determine robustness of the findings.
Randomization	N/A, groups were determined based on diagnostic routine.
Blinding	Sample measurements and data analysis was performed blinded to the diagnostics of the participants.

Reporting for specific materials, systems and methods

We require information from authors about some types of materials, experimental systems and methods used in many studies. Here, indicate whether each material, system or method listed is relevant to your study. If you are not sure if a list item applies to your research, read the appropriate section before selecting a response.

Materials & experimental systems

n/a	Involved in the study	
[x]	[ ]	Antibodies
[x]	[ ]	Eukaryotic cell lines
[x]	[ ]	Palaeontology and archaeology
[x]	[ ]	Animals and other organisms
[ ]	[x]	Clinical data
[x]	[ ]	Dual use research of concern
[x]	[ ]	Plants

Methods

n/a	Involved in the study	
[x]	[ ]	ChIP-seq
[x]	[ ]	Flow cytometry
[x]	[ ]	MRI-based neuroimaging

Clinical data

Policy information about clinical studies

All manuscripts should comply with the ICMJE guidelines for publication of clinical research and a completed CONSORT checklist must be included with all submissions.

Clinical trial registration	(NCT03174938)
Study protocol	Bio-FINDER-2 cohort
Data collection	Participants were recruited at Skåne University Hospital and the Hospital of Ängelholm in Sweden and enrolled between April 2017 and March 2022.
Outcomes	Primary outcome was trajectories of all included fluid biomarkers against SILA-based amyloid- and tau-PET positivity duration. Secondary outcomes were changes in slope for the CSF biomarkers and behavior of specific epitope forms for both CSF and plasma biomarkers.

Plants

Seed stocks	*Report on the source of all seed stocks or other plant material used. If applicable, state the seed stock centre and catalogue number. If plant specimens were collected from the field, describe the collection location, date and sampling procedures.*
Novel plant genotypes	*Describe the methods by which all novel plant genotypes were produced. This includes those generated by transgenic approaches, gene editing, chemical/radiation-based mutagenesis and hybridization. For transgenic lines, describe the transformation method, the number of independent lines analyzed and the generation upon which experiments were performed. For gene-edited lines, describe the editor used, the endogenous sequence targeted for editing, the targeting guide RNA sequence (if applicable) and how the editor was applied.*
Authentication	*Describe any authentication procedures for each seed stock used or novel genotype generated. Describe any experiments used to assess the effect of a mutation and, where applicable, how potential secondary effects (e.g. second site T-DNA insertions, mosiacism, off-target gene editing) were examined.*
